# Cognitive Reserve and Gait Decline Across the Parkinson’s Spectrum: Evidence from PPMI Longitudinal Cohorts

**DOI:** 10.1093/geroni/igaf122.3733

**Published:** 2025-12-31

**Authors:** Han-yun Tseng

**Affiliations:** University of Colorado, Aurora, Colorado, United States

## Abstract

**Background:**

Cognitive reserve has been linked to slower decline in mobility and daily function in Parkinson’s disease (PD), but its role across the prodromal-to-diagnosed spectrum, and it shifts over time, remains unclear.

**Methods:**

We analyzed longitudinal data from the Parkinson’s Progression Markers Initiative (PPMI; baseline N = 3,971; Mage = 62.4 years, 42% female) across four annual visits. Participants included healthy controls (HC; n = 331), prodromal subgroups (hyposmia, RBD, LRRK2 carriers; n = 2,190), and early PD (≤ 2 years from diagnosis; n = 1,450). Mixed-effects models tested between-person (average Montreal Cognitive Assessment [MoCA]) and within-person (time-specific deviations) effects on gait impairment (PIGD), controlling for age, sex, and education.

**Results:**

All groups showed significant gait decline over four years. Higher between-person MoCA predicted slower decline, whereas within-person deviations did not. Subgroup analyses revealed the strongest protective effect in LRRK2 carriers, with weaker effects in hyposmia and RBD. In PD, the reserve effect was attenuated but remained present. Growth models showed significant slope-slope coupling between cognition and gait decline (r = -.24, p < .01), evident when four visits were modeled.

**Conclusions:**

Cognitive reserve helps preserve mobility even before PD diagnosis, but once PD manifests, cognitive and motor decline become closely intertwined This stage-specific shift marks the point where resilience gives way to vulnerability, aligning with biopsychosocial and mind-body frameworks that guide intervention in aging and neurodegeneration. Clinically, findings underscore stratified prevention that harnesses reserve before diagnosis and multimodal interventions that address cognitive–motor interdependence once PD manifests.

